# Paraoxonase-1 and -3 Protein Expression in the Brain of the Tg2576 Mouse Model of Alzheimer’s Disease

**DOI:** 10.3390/antiox10030339

**Published:** 2021-02-24

**Authors:** Jose Gregorio Salazar, Judit Marsillach, Ingrid Reverte, Bharti Mackness, Michael Mackness, Jorge Joven, Jordi Camps, Maria Teresa Colomina

**Affiliations:** 1 Department of Psychology and Research Center for Behavioral Assessment (CRAMC), Universitat Rovira i Virgili, 43007 Tarragona, Spain; salazarjg@ula.ve (J.G.S.); ingrid.reverte@uniroma1.it (I.R.); 2 Research in Neurobehavior and Health (NEUROLAB), Universitat Rovira i Virgili, 43201 Reus, Spain; 3 Departamento de Toxicología y Farmacología, Facultad de Farmacia y Bioanálisis, Universidad de Los Andes, Mérida 5101, Venezuela; 4 Unitat de Recerca Biomèdica, Hospital Universitari de Sant Joan, Institut d’Investigació Sanitària Pere Virgili, Universitat Rovira i Virgili, 43201 Reus, Spain; jmarsi@uw.edu (J.M.); jjoven@grupsagessa.com (J.J.); 5 Department of Environmental and Occupational Health Sciences, University of Washington, Seattle, WA 98105, USA; 6 Department of Physiology and Pharmacology, Sapienza University of Rome, 00185 Rome, Italy; 7 Santa Lucia Foundation (IRCCS Fondazione Santa Lucia), 00179 Rome, Italy; 8 Miami Platja, 43892 Tarragona, Spain; bharti.mackness@gmail.com (B.M.); mike.mackness@gmail.com (M.M.)

**Keywords:** paraoxonases, oxidative stress, Alzheimer’s disease, brain, Tg2576 mice, astrocytes, hippocampus, amyloid-β, microglia, neurons

## Abstract

Background: Brain oxidative lipid damage and inflammation are common in neurodegenerative diseases such as Alzheimer’s disease (AD). Paraoxonase-1 and -3 (PON1 and PON3) protein expression was demonstrated in tissue with no *PON1* or *PON3* gene expression. In the present study, we examine differences in PON1 and PON3 protein expression in the brain of a mouse model of AD. Methods: we used peroxidase- and fluorescence-based immunohistochemistry in five brain regions (olfactory bulb, forebrain, posterior midbrain, hindbrain and cerebellum) of transgenic (Tg2576) mice with the Swedish mutation (KM670/671NL) responsible for a familial form of AD and corresponding wild-type mice. Results: We found intense PON1 and PON3-positive staining in star-shaped cells surrounding Aβ plaques in all the studied Tg2576 mouse-brain regions. Although we could not colocalize PON1 and PON3 with astrocytes (star-shaped cells in the brain), we found some PON3 colocalization with microglia. Conclusions: These results suggest that (1) PON1 and PON3 cross the blood–brain barrier in discoidal high-density lipoproteins (HDLs) and are transferred to specific brain-cell types; and (2) PON1 and PON3 play an important role in preventing oxidative stress and lipid peroxidation in particular brain-cell types (likely to be glial cells) in AD pathology and potentially in other neurodegenerative diseases as well.

## 1. Introduction

Alzheimer’s disease (AD) is the most frequent form of dementia in the elderly, contributing to 60–70% of all dementia cases [1]. It is characterized by the progressive mental deterioration of functions at several cognitive domains, while neuropathological signs gradually build up in several cortical regions of the brain [2]. One of the most prominent and first-appearing neuropathological signs of AD is the presence of amyloid-β (Aβ) plaques. These senile plaques consist of a central core of amyloid-β-insoluble 39–42 residue peptide fragments, surrounded by astrocytes, microglia, and nearby neurons. Aβ in its oligomeric form is toxic and induces oxidative stress, while Aβ aggregates complexed with the redox metal ion copper (Cu) maintain their ability to increase hydroxyl radical formation [3]. There are currently several AD mouse models based on human, genetic, and early-onset forms of AD. The Swedish Tg2576 transgenic mouse is one of the most used AD mouse models. This mouse overexpresses a mutant form of human *APP* (isoform 695), in addition to containing the Swedish mutation (KM670/671NL), leading to an increase in Aβ 1-40 and 1-42 fragments, and the formation of Aβ plaques in the brain [4]. In addition to the high-level expression of the mutant Aβ peptide, Tg2576 mice show a behavioral phenotype characterized by spatial-memory deficits at 9 months of life and beyond, and an increase in microglial density and the number of Aβ plaques in the frontal-, temporal-, and entorhinal-cortex, and hippocampus brain regions. Aged Tg2576 mice also show brain-oxidative lipid damage surrounding Aβ deposits [5].

The paraoxonase (PON) family of enzymes comprises three members, paraoxonase-1 (PON1), -2 (PON2), and -3 (PON3). In humans, *PON1* and *PON3* are expressed in the liver and at lower levels in the kidneys, and their protein products are mainly found in circulating high-density lipoproteins (HDLs) [6,7,8] and the endoplasmic reticulum of intestinal cells [9]. PON3 protein expression was also demonstrated in the mitochondria of selected tissue types [10]. HDLs could act as delivery carriers of PON1 (and likely PON3) from the liver to tissue where its activity is needed on the basis of reports of PON1 transfer to membranes [11,12,13], and the presence of PON1 and PON3 in certain tissue with no demonstrated *PON1* and *PON3* gene expression [14,15,16,17]. All PONs are polymorphic enzymes, with certain polymorphisms affecting their activity or protein expression (reviewed in [18,19]). The physiological roles of these enzymes are still uncertain, although it is accepted that they are lactonases with broad substrate specificity [20,21]. The three PONs are potent antioxidant, anti-inflammatory, and anti-apoptotic enzymes. PON1 and PON3 also participate in HDL-mediated cholesterol efflux from macrophages in a mechanism that involves the ATP-binding cassette transporter A-I (ABCA1) receptor, contributing to an important atheroprotective role [22,23,24,25]. Alterations in circulating plasma PON1 levels are reported in a variety of oxidative stress-related diseases. These include cardiovascular disease, chronic renal failure, HIV infection, metabolic syndrome, chronic liver impairment, and AD [19,26,27].

Substantial evidence indicates that oxidative stress is involved in the pathogenesis and progression of AD [28,29,30]. In monomeric form, Aβ peptides have antioxidant activity; however, when Aβ peptides form aggregates, they exhibit pro-oxidant activity, thus promoting oxidative stress [31]. In AD, lipid peroxidation seems to be a major contributor to oxidative stress damage due to the brain’s high oxygen consumption and the high content of polyunsaturated fatty acids [32]. The presence of oxidative stress products was shown in brain regions with AD histopathologic alterations [33]. Interestingly, brain antioxidant enzyme activity significantly increases in areas of lipid peroxidation, suggesting a compensatory response to increased oxidative stress [34]. Although there is no *PON1* or *PON3* gene expression in the brain [8,35,36,37], we identified PON1 and PON3 protein expression in the white-matter brain areas of healthy C57BL/6J mice [16]. Thus, PON1 and PON3 could play an important role in modulating brain oxidative stress during AD progression. 

The aim of the present study was to examine differences in PON1 and PON3 protein expression in the brain of a mouse model of AD. Our hypothesis was that PON1 and PON3 are somehow transferred from circulation to the brain whenever their antioxidant activities are needed, explaining their presence in brain areas with no *PON1* or *PON3* gene expression [8,35,36,37]. Thus, during AD progression, the protein presence of PON1 and PON3 increases in brain regions with high oxidative stress, which was demonstrated to coincide with the increased presence of Aβ plaques. We found very intense expression of PON1 and PON3 exclusively surrounding Aβ plaques and in star-shaped cells located around those areas.

## 2. Materials and Methods 

### 2.1. Animals

Male transgenic (Tg2576) mice with the Swedish mutation (KM670/671NL), responsible for a familial form of AD, were obtained from Taconic Europe (Lille Skensved, Denmark; strain name: B6;SJL-Tg(APPSWE)2576Kha). Corresponding wild-type (WT) mice were generated by crossing parental APPSwe heterozygous male mice (Taconic Europe) with C57BL6/SJL female mice (Charles River, Barcelona, Spain). For the proposed experiments, mice were genotyped by PCR using DNA extracted from mouse tail at 2 months of age, and separated according to their gender and genotype. Mice were maintained in an animal room with standard conditions: a light/dark cycle of 12:12 h, a temperature of 22 ± 2 °C, and a relative humidity of 50 ± 10%. All mice had food (standard chow diet, Panlab, Barcelona) and drinking water available ad libitum. The experimental protocol was approved by the Animal Care and Use Committee of the Universitat Rovira i Virgili (Tarragona, Spain).

At 18 months of age, 2 Tg2576 and 2 WT mice were sacrificed. After being anesthetized (10 mg/kg xylazine and 100 mg/kg ketamine, i.p.), mice received intracardiac perfusions containing 0.9% NaCl, followed by 4% paraformaldehyde (PFH). Brains were carefully removed, placed in 4% PFH at 4 °C for 2 h, sectioned in coronal sections by adult-mouse-brain slicer matrix BSMAS001-1 (Zivic instruments, Pittsburgh, PA, USA) and the following regions were separated: olfactory bulb, forebrain, posterior midbrain, hindbrain, and cerebellum. These brain regions were then dehydrated and embedded in paraffin, and 2 cm thickness coronal brain sections were obtained for all histological analyses.

### 2.2. Antibodies

Polyclonal rabbit anti-mouse PON1 and PON3 antibodies were obtained as previously described [16] using peptides derived from specific sequences of mature PON1 or PON3. These antibodies were generated against peptides specific to each PON protein and tested for specificity to each PON protein by our laboratory [16] and others [8,38]. The other used antibodies were commercially obtained as detailed below.

### 2.3. Peroxidase-Based Immunostaining

Brain sections from each region were deparaffinized and rehydrated using standard protocols. The buffer used throughout the immunostaining protocol was 20 mM Gly, 100 mM PBS pH 7.4. Rehydrated sections were rinsed in 0.1% Triton X-100 (Sigma-Aldrich, Barcelona, Spain) in Gly-PBS buffer, and incubated in H_2_O_2_ for 10 min to inactivate endogenous peroxidases. Then, sections were blocked for 30 min in 2% bovine serum albumin (BSA, Sigma-Aldrich) in 0.1% Triton X-100, Gly-PBS buffer. Blocking buffer was carefully removed by tapping the slide on a filter paper prior to the addition of the primary antibodies. Anti-PON1 or anti-PON3 antibodies, diluted 1:300 *v*/*v* in 1% BSA, 0.1% Triton X-100, Gly-PBS buffer, were applied over tissue sections and incubated in a humid chamber overnight at 4 °C. Following a 10 min wash with 0.1% Triton X-100, Gly-PBS buffer, sections were incubated for 1 h with a biotinylated anti-rabbit antibody (Vector Laboratories, Barcelona, Spain), diluted 1:200 *v*/*v* in 1% BSA, 0.1% Triton X-100, Gly-PBS buffer. We used the immunoenzymatic antigen-detection system Vectastain ABC kit followed by the DAB Peroxidase (HRP) Substrate kit (both from Vector Laboratories), yielding an insoluble brown product in areas with antigen–antibody recognition. These two kits were used according to the manufacturer’s instructions. Sections were counterstained with hematoxylin for 30 s, dehydrated using standard procedures, and preserved using DPX resin as mounting media (Sigma-Aldrich). Slides were visualized using an upright widefield microscope (Eclipse 600, Nikon, Barcelona, Spain). Pictures were obtained using the AnalySIS image software system (Soft Imaging System, Munster, Germany). Negative-control slides were stained following the same protocol, but omitting incubation with primary antibodies.

### 2.4. Fluorescence-Based Immunostaining

After removing paraffin and rehydrating sections using standard protocols, brain slides were rinsed in 0.1% Triton X-100, Gly-PBS buffer (the same Gly-PBS buffer as that detailed above). Slides were then blocked for 30 min in blocking buffer (2% BSA in 0.1% Triton X-100, Gly-PBS buffer). After removing the blocking buffer by tapping on a filter paper, slides were incubated overnight at 4 °C in a humid chamber with either 1/300 rabbit polyclonal anti-PON1 or 1/300 rabbit monoclonal anti-PON3 primary antibodies, and 1/160 mouse monoclonal anti-glial fibrillary acidic protein GFAP (Calbiochem, Darmstadt, Germany), 1/200 mouse monoclonal antineuronal nuclei NeuN (Millipore, Temecula, CA, USA), 1/100 rat monoclonal anti-neuroectodermal stem-cell marker nestin (Millipore), 1/300 goat polyclonal anti-ionized calcium-binding adapter molecule 1 Iba1 (Santa Cruz Biotechnology, Santa Cruz, CA, USA), and/or 1/500 mouse monoclonal anti-beta amyloid (A8354, Sigma-Aldrich). After two 10 min washes with 0.1% Triton X-100, Gly-PBS buffer, slides were incubated for 3 h at room temperature in a humid chamber with either 1/500 anti-rabbit fluorescein isothiocyanate (FITC)-conjugated (AlexaFluor 488, Invitrogen, Carlsbad, CA, USA) or 1/400 anti-mouse tetramethyl rhodamine isothiocyanate (TRITC)-conjugated (AlexaFluor 568, Invitrogen, Carlsbad, CA, USA) secondary antibodies, and 1/400 anti-goat Cy5-conjugated (Santa Cruz Biotechnology). Primary and secondary antibodies were diluted in 1% BSA, Gly-PBS buffer. The final wash was applied, and sections were mounted with the addition of an antifade reagent (SlowFade Gold Antifade Reagent, Invitrogen, Carlsbad, CA, USA), and stored until observation in a laser-scanning confocal microscope (Nikon NIS-Elements Eclipse TE2000E, Nikon Corp, Tokyo, Japan). Images were adjusted for brightness and contrast using Adobe Photoshop (Adobe Systems, San Jose, CA, USA). Negative-control slides were stained following the same protocol, but omitting incubation with primary antibodies. Additionally, a secondary-antibody negative control was prepared by omitting the secondary instead of the primary antibody in the incubation.

## 3. Results

### 3.1. PON1 and PON3 Peroxidase-Based Immunohistochemistry

**Figure 1:**
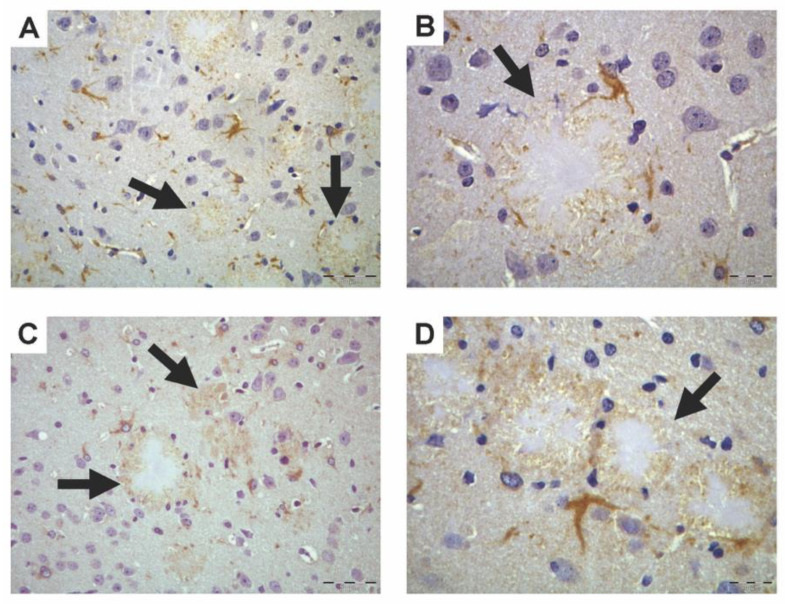
Paraoxonase-1 (PON1) and -3 (PON3) peroxidase-based immunohistochemistry in parietal cortex of the posterior midbrain region of aged Tg2576 mice. (**A**) PON1 immunohistochemistry, 200× magnification; (**B**) PON1 immunohistochemistry, 400× magnification. (**C**) PON3 immunohistochemistry, 200× magnification. (**D**) PON3 immunohistochemistry, 400× magnification. Arrows indicate Aβ plaques.

PON1 and PON3 peroxidase-based immunohistochemistry resulted in moderate staining (brown) localized in brain white-matter fiber tracts, especially in the corpus callosum, and internal and external capsules in both WT and Tg2576 mice. The presence of PON1 and PON3 in WT mice was scarce and mainly located near myelinated tracts, as already described [16]. In the brains of Tg2576 mice, in addition to near-myelinated tracts, PON1 and PON3 staining was highly concentrated around Aβ plaques, located mainly in the hippocampus, and parietal and temporal cortices (Figure 1). These images also demonstrated the presence of intense PON1 and PON3 immunostaining in some morphologically compatible cells with astrocytes, oligodendrocytes, or microglial cells surrounding Aβ plaques. Negative-control slides showed no positive (brown) staining (data not shown).

### 3.2. Fluorescence-Based Immunohistochemistry

#### 3.2.1. PON1 and PON3 Immunofluorescence

**Figure 2:**
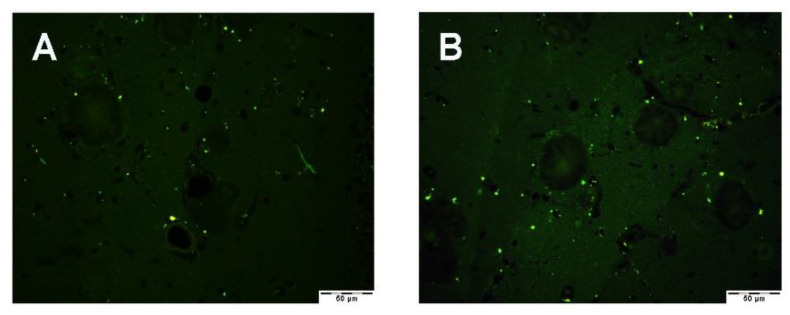

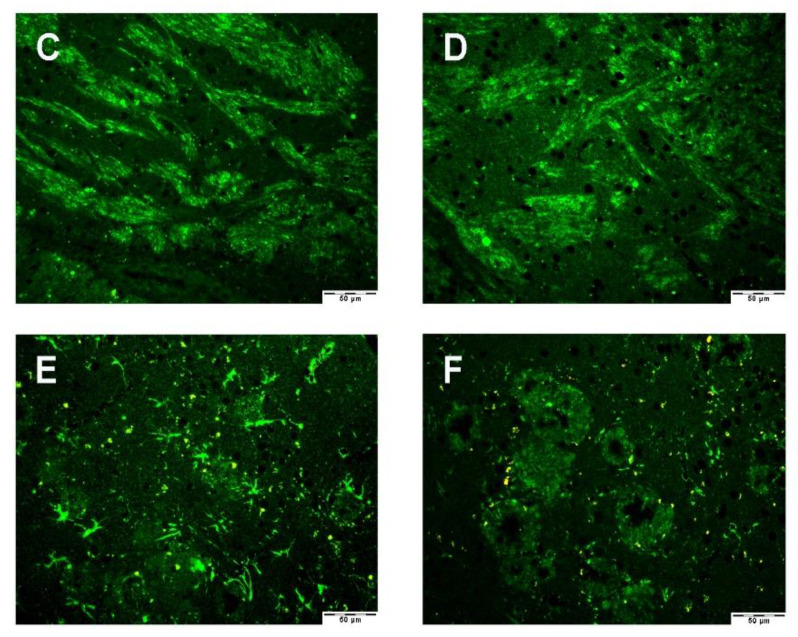
PON1 and PON3 immunofluorescence in parietal cortex of posterior midbrain region. All panels are at 200× magnification. (**A**) PON1 immunofluorescence omitting primary antibody in Tg2576 mice. (**B**) PON1 immunofluorescence omitting secondary antibody in Tg2576 mice. (**C**) PON1 immunofluorescence in WT mice. (**D**) PON3 immunofluorescence in WT mice. (**E**) PON1 immunofluorescence in Tg2576 mice. (**F**) PON3 immunofluorescence in Tg2576 mice.

We used PON1 and PON3 fluorescence-based immunohistochemistry or immunofluorescence to ensure that we could reproduce the results obtained using peroxidase-based immunohistochemistry. Figure 2 shows that PON1 and PON3 immunofluorescence showed background staining in the form of small very bright green precipitates that could be seen in all analyzed slides (negative antibody controls, WT and Tg2576 mice). For the primary- and secondary-antibody negative-control slides, we used Tg2576 mouse-brain sections (Figure 2A,B, respectively). The WT mouse brain showed positive staining (green) in myelinated tracts in both PON1 and PON3 immunofluorescence (Figure 2C,D, respectively), as already reported using peroxidase-based immunohistochemistry [16]. In Tg2576 mice, PON1- and PON3-positive staining (green) was found surrounding Aβ plaques and in cells with a star shape in all brain sections with the presence of analyzed Aβ plaques (Figure 2E,F, respectively). All sections depicted in Figure 2 were obtained from parietal cortex areas of the posterior midbrain region.

#### 3.2.2. Astrocyte–Microglia–Neuron “Triad” Immunofluorescence

**Figure 3:**
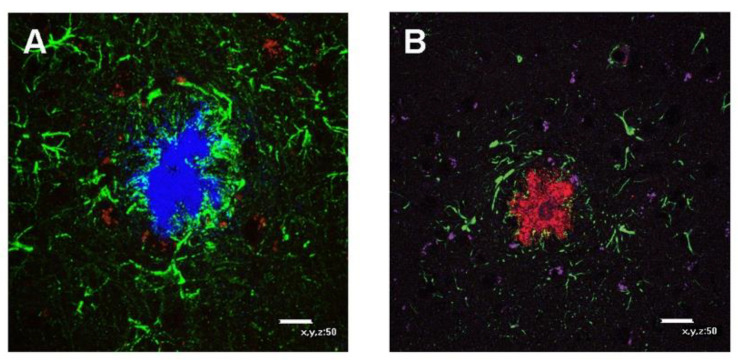
Triple immunofluorescence in parietal cortex of posterior midbrain section of Tg2576 mice. (**A**) Astrocyte (GFAP, green)–microglia (Iba1, red)–Aβ deposition (blue) immunofluorescence, obtained using different lasers and merged. (**B**) Astrocyte (GFAP, green)–neuron (Nestin, purple)–Aβ deposition (red) immunofluorescence obtained using different lasers and merged. All panels are at 400× magnification.

Triple immunofluorescence was conducted to ensure the proper labeling of Aβ plaques and their surrounding cells (astrocytes, microglia, and proliferating neurons). Figure 3A shows that Aβ deposition (in blue) was surrounded by a large number of star-shaped cells corresponding to astrocytes (GFAP, green) and several microglia (Iba1, red). Figure 3B shows that Aβ deposition (in red) was surrounded by astrocytes (GFAP, green) and some proliferating neurons (nestin, purple). GFAP-positive staining in Figure 3 resembled PON1- and PON3-positive staining in Figure 2E,F.

#### 3.2.3. PON1 and Astrocyte–Microglia–Neuron “Triad” Double Immunofluorescence

**Figure 4:**
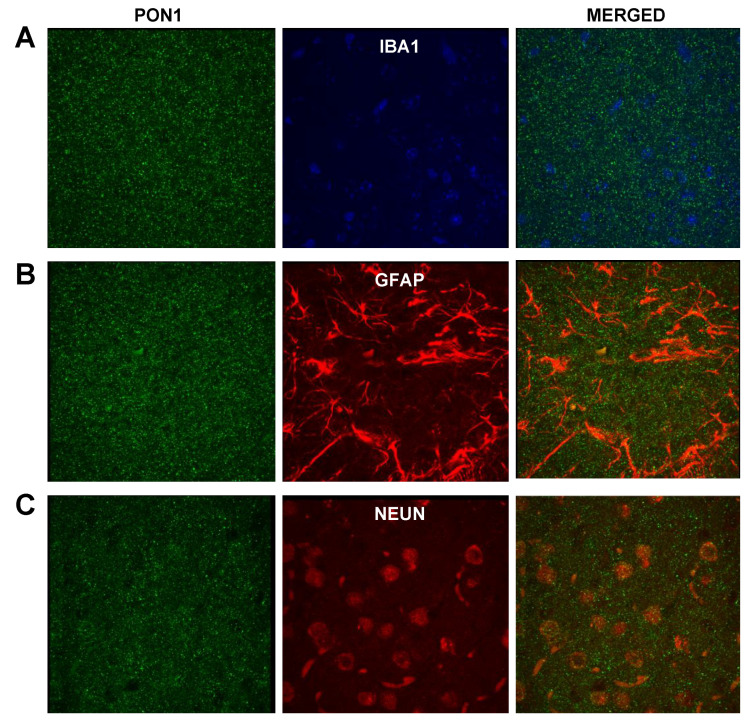
Double immunofluorescence of PON1 and brain cell markers for (**A**) microglia (Iba1), (**B**) astrocytes (GFAP), and (**C**) neurons (NeuN) in sections from Tg2576 mouse-brain temporal cortex; 1000× magnification. Negative-control slides showed no positive (green) staining (data not shown).

We carried out double immunofluorescences targeting PON1 and individual brain cell markers to determine if there was PON1 colocalization with any of the cell types found surrounding Aβ plaques. We focused on the astrocyte–microglia–neuron “triad” and used the following specific cell markers: GFAP for astrocytes, Iba1 for activated microglia, and NeuN for neuronal nuclei. Surprisingly, we did not see colocalization between PON1 and any of the 3 cell markers used in each double immunofluorescence (PON1 and Iba1, PON1 and GFAP, or PON1 and NeuN) (Figure 4). PON1 immunofluorescence showed a significant amount of nonspecific background staining in comparison with that in the obtained results in single PON1 immunofluorescence (Figure 2C,E) that made it difficult to differentiate it from any specific staining. Negative-control slides (for either PON1 primary or the secondary antibody) showed the same small sparse bright green precipitates depicted in Figure 2A and no positive staining (data not shown).

#### 3.2.4. PON3 and Astrocyte–Microglia–Neuron “Triad” Double Immunofluorescence

**Figure 5:**
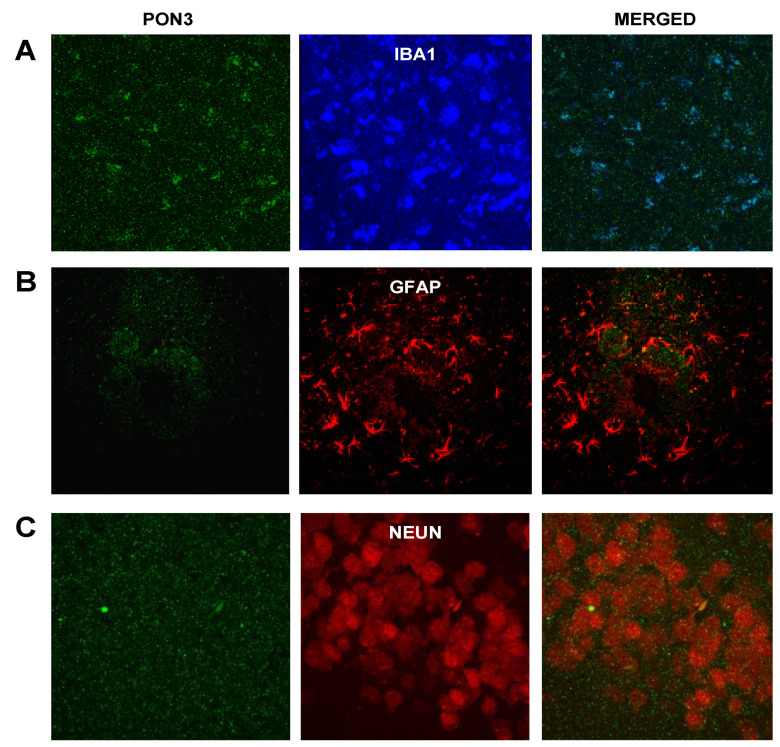
Double immunofluorescence of PON3 and brain-cell markers for (**A**) microglia (Iba1), (**B**) astrocytes (GFAP), and (**C**) neurons (NeuN) in sections from Tg2576 mouse-brain temporal cortex; 1000× magnification. Negative-control slides showed no positive (green) staining (data not shown).

When targeting PON3 and individual components of the astrocyte–microglia–neuron “triad”, we only found some colocalization with microglia (Figure 5A). PON3 colocalized with certain areas of the microglia’s cytoplasm. We did not see colocalization with markers of astrocytes or neurons (Figure 5B,C). As seen with PON1, double PON3 immunofluorescence also showed a significant degree of nonspecific staining. As reported above, the negative-control slides (for either the PON3 primary or secondary antibody) showed similar results to those of Figure 2A and no positive staining (data not shown).

## 4. Discussion

In the present study, the possibility of a differential expression of PON1 and PON3 in the brains of healthy mice and mice with AD pathology was evaluated. Although our results were not conclusive, there was strong evidence of PON1 and PON3 expression surrounding Aβ plaques, and intense positive staining in star-shaped cells that resembled glial cells in areas with an abundance of Aβ plaques. This is the first report describing PON1 and PON3 protein localization in these cells and regions in mouse AD pathology compared with healthy mice.

Although there is no documented *PON1* or *PON3* gene expression in any of the mouse- or human-brain regions [8,35,36,37], an earlier study indicated that PON1 and PON3 proteins are expressed in myelinated fibers in the brain tissue of healthy C57BL/6 mice [16], suggesting that PON1 and PON3 are somehow transferred from blood circulation to the brain. In the same year, another group demonstrated for the first time the presence of PON1 activity in human cerebrospinal fluid [39], with more recent articles reporting decreased cerebrospinal fluid PON1 activity in dementia and AD patients [40,41]. Altogether, this strongly supports the transfer of PON1 and PON3 from blood circulation to the central nervous system. Indeed, in vitro studies showed that small HDL particles are transcytosed across the blood–brain barrier via scavenger receptor class B, type I (SR-B1)-mediated uptake [42,43]. We hypothesize that PON1 and PON3, embedded in discoidal HDLs, would cross the blood–brain barrier and be transferred to these AD pathological brain areas and cells via an unknown mechanism [13]. 

Astrocytes are star-shaped glial cells that have an important role in the synthesis of cholesterol and lipoprotein components, and the assembly of lipoproteins in the brain. Astrocytes also participate in cholesterol efflux to lipoproteins, regulated by ATP-binding cassette transporters A-I (ABCA-1) and G-I (ABCG-1). As plasma HDL-bound PON1 and PON3 participate in cholesterol efflux from macrophages to HDL, the suggested presence of PON1 and PON3 in brain astrocytes does not seem coincidental, and could suggest a role of PON1 and PON3 in astrocyte cholesterol efflux that is highly dysregulated by AD pathology. In fact, the dysregulation of brain-cholesterol homeostasis is associated with neurodegeneration [44] and is considered the third hallmark of AD. However, we were not able to observe the presence of PON1 and PON3 colocalizing with astrocytes. In this regard, and considering the presence of PON1 and PON3 in the myelinated fibers of brain tissue [16], the star-shaped cells in Figure 1 could also be oligodendrocytes. In active demyelinating lesions, oligodendrocytes show a high expression of certain enzymes involved in the antioxidant defense mechanism [45], which would indicate the possibility of PON1 and PON3 being expressed or present in oligodendrocytes, another type of glial cell. In fact, Aβ deposits in the human brain are in close proximity to damaged oligodendrocytes [46]. Oligodendrocytes are highly susceptible to oxidative stress due to their elevated consumption of oxygen and ATP during myelin synthesis, which exposes them to oxidative damage [47], and their low levels of antioxidant enzymes [48]. Thus, exogenous PON1 and PON3 could be delivered to oligodendrocytes to prevent an imbalance in oxidative stress in oligodendrocytes.

Microglia are brain-resident macrophages that interact with all central-nervous-system components, and have an important role in brain development, homeostasis, and disease [49]. Although mainly produced by astrocytes, microglia can also synthesize apolipoprotein E (ApoE), which plays a major role in brain cholesterol transport [50] and cholesterol synthesis. They also express ABCA-1 and participate in cholesterol efflux to ApoE-containing HDL-like particles [51]. In patients with AD, higher numbers of reactive microglia are detected surrounding and phagocytosing Aβ plaques, compared with those in healthy subjects [52]. This neuroprotective “microglial barrier” regulates amyloid compaction and the reduction in neurotoxic fibrils to surrounding neural structures. The dysregulation of lipid metabolism in AD results in the accumulation of cholesterol and decreased Aβ phagocytosis in microglia, leading to the accumulation of Aβ plaques [51]. Our findings of PON3 colocalization in the microglia of mice with AD pathology may indicate the promotion of cholesterol efflux, as speculated in astrocytic PON1 and PON3.

The inflammatory response observed in AD and other neurodegenerative diseases leads to the production of reactive oxygen species (ROS) by activated glial cells (astrocytes, oligodendrocytes, and microglia), contributing to increased levels of oxidative stress reported in neurodegenerative diseases [53,54]. Thus, the suggested localization of PON1 and PON3 in astrocytes or oligodendrocytes, and the observed colocalization of PON3 in microglia indicate a potential antioxidant role of PON1 and PON3 in decreasing levels of ROS and/or preventing lipid peroxidation in these cell types in AD pathology.

## 5. Conclusions

This is a descriptive study that shows for the first time the presence of PON1 and PON3 in Aβ plaques and in star-shaped cells surrounding these Aβ plaques in mice. These results are preliminary, but reinforce the hypothesis that HDLs act as delivery carriers of PON1 and PON3 from the liver to areas of high levels of oxidative stress and inflammation, and suggest that PON1 and PON3 have a yet-to-explore role in astrocytes, microglia, oligodendrocytes, and the AD pathology, which opens a new line of research on the potential role played by PON family members in this and other neurodegenerative diseases.

## Data Availability

Original images are contained within the article.
